# Recent progress and future challenges in algal biofuel production

**DOI:** 10.12688/f1000research.9217.1

**Published:** 2016-10-04

**Authors:** Jonathan B. Shurin, Michael D. Burkart, Stephen P. Mayfield, Val H. Smith

**Affiliations:** 1 Division of Biological Sciences, University of California, San Diego, California, USA; 2 Department of Chemistry and Biochemistry, University of California, San Diego, California, USA; 3 Department of Ecology and Evolutionary Biology, University of Kansas, Kansas, USA

**Keywords:** microbial, biofuel production, algae

## Abstract

Modern society is fueled by fossil energy produced millions of years ago by photosynthetic organisms. Cultivating contemporary photosynthetic producers to generate energy and capture carbon from the atmosphere is one potential approach to sustaining society without disrupting the climate. Algae, photosynthetic aquatic microorganisms, are the fastest growing primary producers in the world and can therefore produce more energy with less land, water, and nutrients than terrestrial plant crops. We review recent progress and challenges in developing bioenergy technology based on algae. A variety of high-value products in addition to biofuels can be harvested from algal biomass, and these may be key to developing algal biotechnology and realizing the commercial potential of these organisms. Aspects of algal biology that differentiate them from plants demand an integrative approach based on genetics, cell biology, ecology, and evolution. We call for a systems approach to research on algal biotechnology rooted in understanding their biology, from the level of genes to ecosystems, and integrating perspectives from physical, chemical, and social sciences to solve one of the most critical outstanding technological problems.

## Introduction

Our present day petroleum reserves are the legacy of phytoplankton growing over hundreds of millions of years. The modern day descendants of these sources of fossil energy have usefully retained the ability to produce the same energy-rich compounds that made their ancestors essential to the development of modern society. The tantalizing possibility that biotechnology may harness the capacity of photosynthetic microorganisms to generate energy by fixing carbon from the atmosphere has stimulated a burst of research activity.

It remains to be seen when and how photosynthetic microbial biofuel production will help solve the conundrum of how we maintain and extend our modern standards of living without further disrupting the environment. Will microbial biofuels prove to be a silver bullet, one element of a broader solution to the energy economy, or perhaps just an expensive lesson in the limits of biotechnology? Microscopic algae offer clear advantages over terrestrial crops in that they grow at far faster rates and can be cultivated on non-arable land and with non-potable water, lessening the pressure placed on existing food production systems.

Here we describe recent progress in understanding the cultivation of microscopic algae for the production of energy. We outline key technical and economic gaps in the pathway toward large-scale commercialization and discuss opportunities for further progress. We argue that realization of the full potential of bioenergy from algae demands a perspective rooted in systems biology that integrates understanding of the genetics, cell biology, physiology, evolution, and ecology of photosynthetic microorganisms. The genetic and physiological origin of traits that determine biochemical composition and growth under variable conditions must be understood in order to optimize strains through classical genetics, breeding, or targeted molecular manipulation of the genome. The interactions between cultivated strains and the diverse assemblages of microbial life that invariably colonize outdoor production ponds or enclosed systems are equally important to commercial success.

## Is algal bioenergy feasible?

Life-cycle analysis has been applied to assessing the plausibility of generating significant quantities of bioenergy from algae at a scale to impact the energy economy and reduce global carbon emissions and to identify targets for advancing commercialization. Capital construction costs, biomass yield and oil content, oil price, and the value of residual biomass products all affect the energy return on energy invested as well as the rate of return on capital investment ^1^. Stephens *et al*. ^2^ modeled the sensitivity of the rate of return to these factors and identified biomass productivity and co-product value as key factors driving commercial potential. They also determined that a number of realistic scenarios led to viable returns on investment, indicating that micro-algal biofuel systems approach profitability and that developing synergies with human or animal food systems, water treatment, or other industries is key to their success. Many potential bottlenecks of the algal biofuel engineering and production pathway have now been identified and studied, including harvesting ^3^, oil extraction ^4^, and conversion to fuel ^5^, and best practices developed. However, many opportunities to improve production efficiency and bring the cost of algal biofuels closer to parity with petroleum lie in the realm of biology.

## What are the challenges to increased biomass yield?

The tremendous increase in agricultural productivity over the last few decades stands on the shoulders of millennia of crop plant domestication. Algae are at the very beginning of this process, with no deep body of knowledge of their domestication accumulated from a long and detailed history of breeding and cultivation. The ancestors of present-day crops could not generate the yields we now enjoy or come close to sustaining the current global population. The challenge of algae is to accelerate this domestication process to the point where algae can play a meaningful role in the global carbon cycle and energy economy within the next decade.

Algae are a polyphyletic group of the oldest and most diverse organisms on earth; therefore, they present a rich array of genetic variation on which domestication can act. Targets of genetic and metabolic manipulation include genes and pathways involved in producing primary and secondary metabolites, carbon fixation, and light capture, among many others ^6, 7^. For example, manipulation of gene expression through metabolic engineering can alter both the composition and the quantities of lipids, which are key energy molecules of both microalgae and fossil fuels, like petroleum. However, the regulatory networks that determine the rate of formation of carbon storage molecules like starches and lipids, and the composition of fatty acids, remain poorly elucidated.

## Recent progress in understanding algal genetics

Over the last decade, our ability to sequence and assemble genomes of almost any algal species has accelerated to the point where knowledge about algal genomes is no longer a limiting factor for algal domestication. What is limiting is our understanding of the functioning of algal genes and genetic interaction networks. However, domestication of crops is never just about what genes are in a plant; it is also about how these genes are regulated and interact, especially in traits resulting from many genes acting in concert. It is also important to point out that high-yielding crops did not result from any specific understanding of genes or genetic regulatory networks. Rather, selection on traits over time eventually produced the correct set of genes and gene interactions required to achieve the desired phenotypes. Today, our understanding of algal genomes, coupled with high-throughput screening methods, including rapid genome sequencing, allows us to rapidly associate genotypes with phenotypes. For example, the phenotypic variation observed in distinct wild-type isolates of the green algae *Chlamydomonas reinhardtii* can now be quickly correlated with the genomic sequence of these isolates to correlate genomic DNA sequence changes with desirable phenotypes ^8^. Recent advances in uncovering the genetic basis of important traits may allow us to attain high-yielding domesticated algae species in a fraction of the time it took us to breed traditional crops. In the future, as we gain a better understanding of algal gene function and improve computational methods to both understand and predict gene interactions, we will continue to increase the rate at which we improve algal productivity.

## What co-products can be made?

Algae are simple biological machines that convert sunlight, carbon dioxide, and minerals into biomass, with primary products consisting of proteins, lipids, and carbohydrates. These molecules have utility as food or feed, lipids for biofuels, or even proteins for industrial or medical uses. A combination of algal biology and process development will allow a variety of products to be made in a more efficient and cost-effective manner than is done today with other methods. Algae are presently recognized as ingredients in many foods, but soon we may see larger markets for algae as proteins for animal and human nutrition and as ingredients in functional foods containing vitamins and other nutrients ^9– 11^. In addition, algae have been leveraged for the production of a variety of value-added products such as cosmetic ingredients ^12^, pharmaceutical proteins ^13^, and biobased chemicals ^14, 15^. Some bio-products may be produced more cheaply in algae than by using fermentation in yeast or bacteria ^13^.

## Risks and benefits of genetically engineered algae

The use of metabolic engineering, transgenic technologies, and even synthetic biology to refine algal traits may greatly accelerate the process of domestication to help realize the commercial potential of algae as a source of energy and other products. For instance, Radakovits *et al.* ^6^ sequenced the genome and developed transformation methods for manipulating the lipid synthesis pathways of *Nannochloropsis gaditana*, enabling considerable strain improvements using genetic engineering (GE) technologies. However, genetic modification also generates considerable controversy within science and society at large over concerns about the risks of transgenic technologies to the environment ^16^. Questions about GE organisms center on their potential impacts on human health and the environment. Algae that are cultivated for non-consumptive purposes present less risk for human exposure, but the potential impacts on the environment remain unresolved ^17, 18^. Could transformed algae escape from cultivation, and what is their likely effect on the environment?

Framing the question about the utility of GE algae requires a quantitative assessment of both their advantages as biofuel feedstocks and their potential hazards to the environment. It is possible that algal biofuels cannot become competitive with fossil fuels in a reasonable time frame without the use of GE ^19^. Targets for improving algae bioenergy crops using GE technology include increased lipid content, pathogen resistance, and high-value co-products ^6, 20^. Many of these traits could potentially be introduced using traditional breeding, or mutagenesis and high-throughput screening, but not all traits can be introduced in this way, and breeding is not available in many algal species. GE technologies are a viable, and possibly essential, option for the economic viability of algae as a source of renewable chemicals.

The questions of environmental safety and social acceptance are much harder to address. Many of the traits that we want to engineer into algae, such as high lipid content, seem unlikely to increase their competitive abilities or fitness in the natural environment; however, this inference requires empirical study and validation. One environmental concern is the loss of algal diversity and potential dominance by single GE species or strains in nature. What gene or trait might allow such a species to be constructed, or why such a species would not already have evolved by natural selection alone, is unclear. GE algae may obey the same tradeoffs as strains that evolved by natural selection, but this assumption remains to be shown by experimental analysis. Social acceptance is even harder to predict, as it is shaped by scientific evidence as well as vigorous debate in the public sphere. Developing carbon-neutral algal biofuels with small footprints offers clear environmental benefits, while quantifying the dangers of engineered algae to the environment is a more difficult, but crucial, stage in their development. Clearly, communicating the risks and benefits of GE technology is critical to fostering social acceptance.

Assessing the environmental risks associated with GE algae is a daunting scientific and technical challenge, but it is achievable with careful experimental study of the impact of genetic modification on the dispersion of algae and their interactions with native ecosystems. Burkart ^21^ illustrated this approach in the first outdoor growth trial of the green alga *Acutodesmus dimorphus* transformed with acyl carrier protein thioesterase (to modify fatty acid expression) and green fluorescent protein (as a marker). Both traits were maintained in outdoor cultivation. The transformed strain dispersed overland and could invade experimental algal ecosystems. However, its impact on the productivity, species diversity, and composition of invaded native communities was indistinguishable from that of the wild-type strain. The results show that while genetic modification affected the phenotype of *Acutodesmus* by enhancing fatty acid expression, it had no apparent impact on its ecology. This trial points the way to an informed assessment of the risks and benefits of genetic modification of algae based on simultaneous examination of their potential for cultivation and interactions with natural ecosystems.

## Evolutionary engineering

Domestication of plants and animals throughout human history has resulted from artificial selection on standing genetic variation present in natural populations. Experimental evolution shows that microbes evolve over tens of thousands of generations under selection as new mutations arise and affect fitness ^22^. The phenotypes of microorganisms like algae therefore represent a moving target due to large population sizes, rapid generation times, and the continual influx of new mutations and therefore new traits.

Evolution presents both challenges and opportunities to biofuel production. First, microorganisms may not retain traits acquired through breeding or GE as the growth and harvesting process imposes selection on new mutations that arise or enter the population through horizontal gene transfer. However, selection may also be used to exploit natural variation in populations or communities. Mooij *et al*. ^23^ show that inducing nitrogen limitation during the day imposed selection for algae from a natural community with high carbon storage capacity in the form of lipids or starches. Not all carbon storage compounds are useful as biofuel; however, their experiment demonstrates that the environment can be manipulated in ways that select for specific traits among naturally occurring strains.

## Ecological engineering

Exploiting the capacity of diversity to enhance nutrient capture and conversion to biomass has been proposed as another approach to increasing the productivity of algae ^24^. Stockenreiter ^25^ found that biomass and lipid accumulation increased with the number of species in both experimental and natural algal assemblages. Cultivating diverse species may therefore enhance the uptake of light or nutrients and their incorporation into energy-rich compounds. However, constructing stable or productive communities will likely prove as challenging as maintaining stable phenotypes due to invasion, intrinsic population instabilities, and inevitable environmental variation.

Even the simple environments of biofuel production facilities contain a bewildering diversity of microorganisms. In an algal production pond sampled over a complete annual cycle, Beyter *et al*. ^26^ identified 26,135 prokaryotic and 9,631 eukaryotic sequences of the 16S and ITS2 regions of the genome, respectively. They found that periods with greater eukaryotic diversity and lower prokaryotic diversity corresponded to more stable and productive communities, consistent with ecological theory ^27^. The challenge is to usefully harness the capacity of diversity to enhance yield and stability while ensuring the consistent production of valuable compounds. A working knowledge of the interactions that govern the dynamics and productivity of algal communities containing such diversity of species remains elusive.

## Synthesis: systems biology

The problem of harnessing the productive capacity of microbes to convert light, carbon dioxide from the atmosphere, and nutrients from the environment into energy-rich compounds to power society illustrates many of the most pressing challenges at the forefront of biotechnology. How does the environment affect the translation of genomes into phenotypes? How do the myriad interactions among microorganisms govern the production of biomass and its fate? What is the capacity of natural or engineered ecosystems to absorb excess carbon from the atmosphere and generate useful biomass? These questions span the range of organizational levels of biology and spatial scales from molecules to the biosphere.

The phenotypes that determine the capacity of algae to produce bioenergy are under the control of complex gene networks, as well as aspects of the physical and environmental environment. For instance, Radakovits *et al*. ^6^ identified the gene pathways involved in the synthesis of triacylglycerides (TAGs) in *Nannochloropsis* and showed stable transformation of cells using endogenous promoters. They established links from the genome to the phenotype using genomics, transcriptomics, proteomics, and metabolomics. However, whether transformations at the lipid synthesis pathways are stable against evolutionary decay through mutation or selection imposed by the cultivation environment, or ecological invasion of wild pests and competitors, remains to be seen.

Meeting the technical challenge of generating power and fuels from contemporary, rather than fossil, primary producers demands an approach rooted in systems biology that has a rich understanding of genetics, cell biology, evolution, and ecology. The synthesis of these diverse fields, organized around the common goal of sustaining our quality of life without degrading the environment and threatening the natural systems on which human life depends, should be a paramount research priority. It demands integration across levels of organization from molecules to genes to cells to ecosystems and therefore collaboration among biologists, chemists, and engineers as well as economists and social scientists. Such efforts challenge scientists to break down disciplinary boundaries in order to solve the most urgent challenges facing society.
